# Profiles of opportunistic infections in people living with HIV followed at the Military Hospital of Kinshasa Reference (Camp Kokolo), DRC

**DOI:** 10.1186/1742-4690-9-S1-P146

**Published:** 2012-05-25

**Authors:** NE Kamangu, NH Situakibanza, LG Mvumbi, IL Kakudj, TD Tshienda, TG MESIA

**Affiliations:** 1 Université de Kinshasa, Kinshasa, Congo

## Introduction

In the Democratic Republic of Congo (DRC), Opportunistic Infections (OI) are still a major problem in the care of People Living with HIV (PLHIV). Through the military medical program, few data are available regarding the treatment and prevention of OI. Because of their high mobility and the environment in which they live, the security forces may be considered a population at risk. This study aims to determine the profile of opportunistic infections encountered in PLHIV supported the Military Hospital of Kinshasa Reference (Camp Kokolo / DRC).

## Methodology

This study was conducted at the Military Hospital of Kinshasa Reference (HMRK). It is a literature review that focused on issues of HIV adult patients 18 years followed in the course of January 1^st^ to December 31^st^ 2010. The elements of interest were: age, sex, infections diagnosed. Records that did not contain all of these data were not included.

## Results

Tuberculosis (43.2%), candidacies (oral (16.7%), vaginal (2.3%) and esophagus (1.5%)) prurigo (15.9%), pneumonia (11, 4%), malaria (10.6%), herpes zoster (9.8%), isosporiasis (5.3%), condyloma (3.8%) and salmonella (3.8%) infections were found in patients with HIV attending HMRK.

## Conclusion and recommendations

Tuberculosis, oral candidacies, prurigo, pneumonia and malaria are the opportunistic infections found in these patients. We believe it is necessary to systematize the search for co-association of tuberculosis in any PLHIV.

